# Non-Destructive Detection of the Freshness of Air-Modified Mutton Based on Near-Infrared Spectroscopy

**DOI:** 10.3390/foods12142756

**Published:** 2023-07-20

**Authors:** Peilin Jin, Yifan Fu, Renzhong Niu, Qi Zhang, Mingyue Zhang, Zhigang Li, Xiaoshuan Zhang

**Affiliations:** 1 College of Information Science and Technology, Shihezi University, Shihezi 832000, China; jinpeilin@stu.shzu.edu.cn (P.J.); nrz1994@stu.shzu.edu.cn (R.N.); zhangqq026@163.com (Q.Z.); zhangmy219@163.com (M.Z.); 2 Beijing Laboratory of Food Quality and Safety, College of Engineering, China Agricultural University, Beijing 100083, China; n897593557@163.com

**Keywords:** near-infrared spectroscopy, mutton quality detection, texture parameters, modified atmosphere packaged

## Abstract

Monitoring and identifying the freshness levels of meat holds significant importance in the field of food safety as it directly relates to human dietary safety. Traditional packaging methods for lamb meat quality assessment present issues such as cumbersome operations and irreversible damage. This research proposes a quality assessment method for modified atmosphere packaging lamb meat using near-infrared spectroscopy and multi-parameter fusion. Fresh lamb meat quality is taken as the research subject, comparing various physicochemical indicators and near-infrared spectroscopic information under different temperatures (4 °C and 10 °C) and different modified atmosphere packaging combinations. Through precision parameter comparison, rebound and TVB-N values are selected as the modeling parameters. Six spectral preprocessing methods (multi-scatter calibration, MSC; standard normal variate transformation, SNV; normalization; Savitzky–Golay smoothing, SG; Savitzky–Golay 1 derivative, SG-1st; and Savitzky–Golay 2 derivative, SG-2nd), and three feature wavelength selection methods (competitive adaptive reweighted sampling, CARS; successive projections algorithm, SPA; and uninformative variable elimination, UVE) are compared. Partial least squares (PLS) and support vector machine (SVM) are used to construct prediction models for chilled fresh lamb meat quality. The results show that when rebound is used as a parameter, the SG-2nd-SPA-PLSR model has the highest accuracy, with a determination coefficient R^2^p of 0.94 for the prediction set. When TVB-N is used as a parameter, the MSC-UVE-SVM model has the highest accuracy, with an R^2^p of 0.95 for the prediction set. In conclusion, the use of near-infrared spectroscopic analysis enables rapid and non-destructive prediction and evaluation of lamb meat freshness, including its textural characteristics and TVB-N content under different modified atmosphere packaging. This study provides a theoretical basis and technical support for further encapsulating the models into portable devices and developing portable near-infrared spectrometers to rapidly determine lamb meat freshness.

## 1. Introduction

With the continuous improvement of people’s living standards and the abundant supply of meat in the market, people have higher requirements for the quality of meat. They are also more concerned about the nutritional value, taste, texture, and appearance of meat, as well as its convenience, healthiness, and safety. However, consumers can only rely on visual and olfactory judgments to select high-quality meat [1,2]. Sheep meat is prone to microbial contamination during storage, transportation, and sales, leading to spoilage and the loss of its edibility and commercial value. Modified atmosphere packaging technology can partially compensate for the limitations of vacuum packaging and further delay microbial-induced spoilage [3]. Traditional detection methods often require experienced professionals to perform inspection, which is time-consuming and destructive to the samples, thus reducing detection efficiency and no longer meeting the current industrial development requirements [4,5]. The non-destructive assessment of lamb meat quality using near-infrared spectroscopy and multi-parameter fusion in modified atmosphere packaging can avoid the product damage caused by traditional destructive testing and achieve accurate and rapid real-time detection of the products [6,7,8].

Near-infrared spectroscopy (NIRS) detection technology has been a rapidly developing spectral analysis technique in recent years. It offers advantages such as non-destructiveness, environmental friendliness, speed, and high accuracy, and has been widely applied in food safety testing [9,10]. Fresh meat, as the target of detection, is complex in composition, containing numerous chemical components, which results in diverse near-infrared spectroscopic characteristics. Therefore, many researchers have adopted various spectral preprocessing methods or algorithms. For example, in terms of Savitzky–Golay (SG), convolution smoothing, and derivative methods [11,12,13], the most common approach for building prediction models based on spectral data is to use the entire spectrum information composed of hundreds or even thousands of wavelengths. However, this can introduce interference from a large amount of redundant information, leading to poor model accuracy and unsatisfactory predictive performance. The effective selection of feature wavelengths is a good method for simplifying the workload and improving efficiency, such as regression coefficients, competitive adaptive reweighted sampling (CARS), Fisher Linear Discriminant Transformation, and genetic algorithms [14,15,16].

Previous studies have shown that many scholars have applied near-infrared spectroscopy technology to meat quality detection, including pork and lamb [17,18,19]. Li et al. used Fourier transform near-infrared (FT-NIR) spectroscopy combined with fuzzy clustering algorithms to identify the storage time of pork [20]. Zhang et al. proposed a model based on ensemble learning to predict the TVB-N content of pork using near-infrared spectroscopic information [21]. Lintvedt et al. [22] used near-infrared technology to determine the fat composition in salmon fillets, but there have been few studies on lamb meat quality detection in modified atmosphere packaging. Low temperature combined with modified atmosphere packaging is considered an important technology for maintaining fresh meat quality standards and extending shelf life [23]. It is cost-effective, has a high safety factor, and does not alter the taste of the food. Correct modified atmosphere packaging methods are essential to ensure the quality and prolong the shelf life of meat products [24]. The gas composition commonly used in meat packaging is 20–30% CO_2_ and 70–80% O_2_ [25].

During the process of meat spoilage, enzymes, and bacteria produce a class of substances containing alkaline nitrogen, known as volatile basic nitrogen (TVB-N) [26]. As fresh meat deteriorates, the content of TVB-N gradually increases, making it widely recognized as an important indicator for assessing meat freshness [27]. However, traditional methods for determining TVB-N content mainly rely on the Kjeldahl method [28], which has several drawbacks including low efficiency, time-consuming procedures, and sample destruction. Therefore, it is not suitable for on-site testing requirements in the modern meat processing industry. Similarly, the textural characteristics of meat [29], such as hardness, rebound, elasticity, adhesiveness, cohesiveness, and chewiness, are also key parameters that determine the overall quality of meat products. Therefore, establishing predictive models for the texture characteristics and TVB-N content of modified atmosphere packaged lamb meat is of significant importance for meat quality detection.

**Figure 1:**
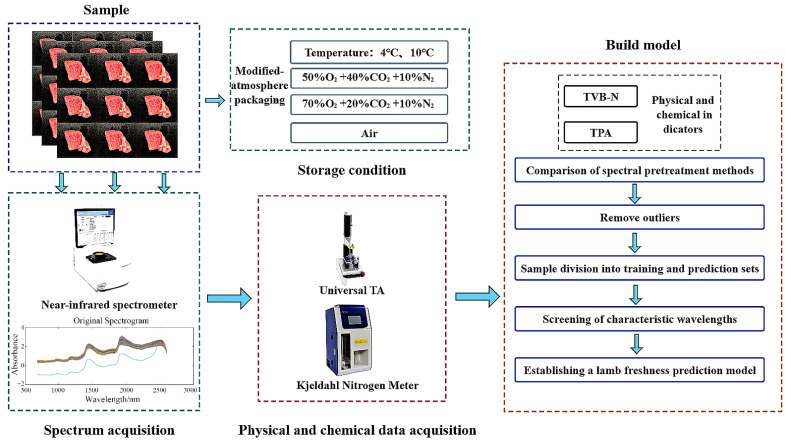
Flow chart of the test and modeling process.

Therefore, this study focuses on controlled atmosphere lamb meat stored at temperatures of 4 °C and 10 °C [30]. The quality attributes of the lamb meat, including textural characteristics and TVB-N content, were determined using physical and chemical tests. The original spectral information was subjected to various preprocessing techniques using chemometrics. The competitive adaptive reweighted sampling algorithm (CARS), the uninformative variable elimination method (UVE), and the successive projections algorithm (SPA) were employed to select the optimal wavelengths. Prediction models were established using partial least squares (PLS) regression and support vector machines (SVM). Ultimately, a lamb meat quality detection model based on selected wavelengths from near-infrared spectroscopy and multiple parameters was developed. The experimental and modeling procedures are illustrated in Figure 1. The specific objectives of this study were as follows: (1) to compare the predictive performance of models combining textural characteristics, TVB-N values, and spectral data, including both original spectra and six different spectral preprocessing methods, to determine the model inputs; (2) to compare three novel methods for extracting feature wavelengths from near-infrared spectra; and (3) to establish lamb meat quality prediction models based on feature wavelengths extracted using the promising methods. These models would provide a foundation for the quality assessment of meat products using portable near-infrared spectroscopy devices.

## 2. Materials and Methods

### 2.1. Experimental Methods

The tools and cutting board used for portioning the lamb meat were wiped with sterilized ethanol. Sterile knives were used to slice the hind leg lamb meat into approximately 2.5 cm thick samples. A total of approximately 180 lamb meat samples were obtained and packed in sealed bags. The bags were then placed in a refrigerated storage box maintained at a temperature range of 4 to 8 °C. Afterward, the samples were transported to the laboratory. Upon arrival, the lamb meat samples were grouped according to the conditions specified in Table 1, with 30 pieces of lamb meat allocated to each group. The samples were temporarily stored under the respective conditions without compression. The measurements were conducted continuously for a duration of 10 days.

**Figure 2:**
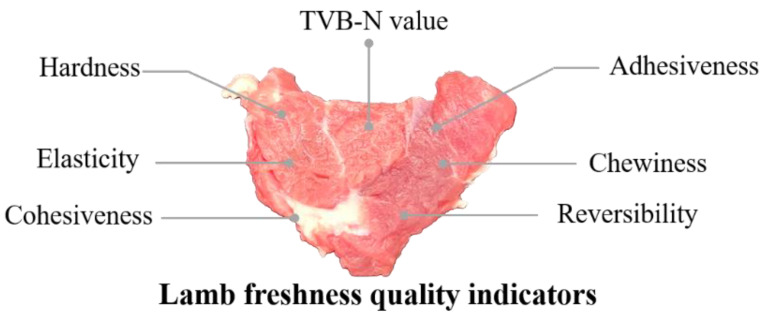
Freshness evaluation index.

During the experiment, under different temperature conditions (4 °C and 10 °C) and various modified atmosphere packaging combinations, the tested samples were taken out daily for a period of 10 days and allowed to equilibrate at room temperature (approximately 20 °C). This step facilitated the sufficient evaporation of surface moisture from the samples and ensured stable contact of the meat surface with air, thereby reducing errors during spectral data collection [31]. Subsequently, the lamb meat samples were subjected to texture analysis using a texture analyzer, and a portion of the lamb meat was taken for TVB-N content analysis. Finally, the samples were placed in a near-infrared spectroscopy instrument for spectral data collection. The evaluation indices for lamb meat freshness obtained are depicted in Figure 2.

### 2.2. NIR Spectral Data Acquisition

The near-infrared spectroscopy instrument was powered on and allowed to preheat for a minimum of 30 min. After performing resolution calibration, the finely ground lamb meat was densely packed into the sample cup provided with the instrument. Care was taken to ensure that the meat was uniformly distributed at the bottom of the cup without any air bubbles. A customized standard white plate was placed over the opening of the sample cup to avoid any spectral data anomalies caused by human manipulation or external environmental factors. The sample cup was then placed on the instrument’s sample tray, and the scanning process was initiated to obtain spectral data. A total of 180 samples were prepared, and each sample was scanned five times, resulting in a total of 900 spectral data records.

### 2.3. Determination of Physical and Chemical Indexes

#### 2.3.1. Determination of Texture Characteristics

The texture analysis was conducted using the texture analyzer from SMS Company, UK. There are various evaluation methods for texture, and the commonly used method is texture profile analysis (TPA), which simulates the chewing movement in the oral cavity [32]. The sample was compressed twice during the test, and the test process was connected to a computer, which provided an interface for outputting the texture test curve. A P5 probe was selected, with a testing speed range of 0.1–10 mm per second (mm/s), testing distance accuracy of 0.1 mm, and testing force accuracy of 0.1 g. Three different points on each sample were selected for measurement, resulting in a total of 540 data records.

With increasing storage time, lamb meat exhibits increased hardness, cohesiveness, and chewiness due to protein degradation, moisture loss, and changes in connective tissues [33]. However, elasticity and adhesiveness tend to decrease.

#### 2.3.2. TVB-N Content Determination

The method for determining TVB-N in this experiment followed the guidelines outlined in *GB 5009.228-2016* [34]. Approximately 10.00 g of ground lamb meat sample was weighed and transferred to a 50 mL centrifuge tube. Then, 40 mL of 0.6 molar per milliliter (mol/L) perchloric acid solution was added to the tube to make it up to volume. The tube was centrifuged at 4000× *g* revolutions per minute (rpm) for 10 min in a centrifuge machine. Subsequently, 20 mL of the supernatant was transferred to a digestion tube in a Kjeldahl nitrogen analyzer for the determination of TVB-N content. Each sample was tested three times, and the average value was calculated. According to the regulations specified in *GB 2707-2016* [35] “National Food Safety Standard for Fresh (Frozen) Livestock and Poultry Products”, the TVB-N content of chilled lamb meat with first-grade freshness should not exceed 15 milligrams per 100 g of lamb meat (15 mg/100 g).

### 2.4. Statistical Analysis

#### 2.4.1. Abnormal Spectral Sample Rejection

In qualitative analysis, samples corresponding to spectral anomalies are commonly referred to as abnormal samples. The Mahalanobis distance (*D_i_*) is calculated for each sample, and an outlier threshold (*D*_th_) is set to identify and remove abnormal samples [36]. The calculation method for the Mahalanobis distance of each sample is as follows:

(1) $$D_{i}^{2}=(t_{i}-\bar{T})M^{-1}(t_{i}-\bar{T}{)}^{\prime }$$

(2) $$\bar{T}{=}^{\left(\sum_{1}^{m}t_{i}\right)}/m$$

where *M* represents the covariance matrix of the principal component scores matrix of the training set spectra, $t_{i}$ denotes the principal component score vector of sample *i*, $\bar{T}$ is the average score matrix of m training set samples, and $D_{i}$ represents the Mahalanobis distance of training set sample *i*.

The formula for calculating the threshold for detecting abnormal samples in the training set is as follows:

(3) $$D_{th}=D_{m}+e\cdot \sigma_{d}.$$

Given the threshold adjustment weight coefficient *e*, where $D_{m}$ and $\sigma_{d}$ represent the average value and standard deviation of the Mahalanobis distances for m samples, if $D_{i}\geq D_{th}$, the *i*th sample in the training set is considered an abnormal sample and is removed. Conversely, if $D_{i}<D_{th}$, it is considered that the spectrum of sample *i* in the principal component space is similar to the others in the training set.

#### 2.4.2. Spectral Preprocessing

Before establishing the prediction model, six different spectral preprocessing methods with distinct effects [37] were employed. Multi-scatter calibration (MSC) effectively eliminates spectral differences caused by varying levels of scattering, thus enhancing the correlation between spectra and data [38]. Standard normal variate transformation (SNV) reduces the gaps and differences between two spectra, resulting in more compact spectra. This transformation helps eliminate differences caused by factors such as particle size, leading to increased consistency among spectra with similar sample properties. Normalization (Nor) scales and shifts spectra proportionally to eliminate the influence of data dimensions, ensuring comparability between spectral indices. It is crucial for establishing the model. [39] Savitzky–Golay smoothing (SG) is a convolutional smoothing technique that suppresses signal fluctuations, thereby reducing spectral noise. Savitzky–Golay’s first-order derivative (SG-1st) and second-order derivative (SG-2nd) also provide some inhibition of signal vibrations, contributing to noise reduction in the spectra.

#### 2.4.3. Selection of the Characteristic Wavelength

To address the issue of redundant spectral bands and noise affecting modeling accuracy within a large number of wavelength points, it is essential to identify wavelength points that contribute significantly to the modeling results. Feature wavelength selection, where a subset of wavelengths replaces the entire spectrum, can be employed. In this study, three feature wavelength selection methods were used: competitive adaptive reweighted sampling (CARS) [40], successive projections algorithm (SPA) [41], and uninformative variable elimination (UVE) [42]. These methods aim to identify the most informative wavelength points for modeling by considering their contributions to prediction accuracy.

#### 2.4.4. Division of the Sample Set

The experimental samples were divided into training and prediction sets using three methods: random partitioning, Kennard–Stone, and SPXY algorithms. In the Kennard–Stone (KS) algorithm, all samples are initially considered candidates for the training set. Samples are selected one by one into the training set based on their distances. The algorithm starts by choosing the two samples with the maximum Euclidean distance and adds them to the training set. Subsequently, for each remaining sample, the Euclidean distance to each known sample in the training set is calculated. The sample with the maximum minimum distance is selected and added to the training set. This process continues until the desired number of samples is reached [43]. The formula for calculating the Euclidean distance is as follows:

(4) $$d_{x}(p,q)=\sqrt{\sum_{j=1}^{N}{\left[x_{p}(j)-x_{q}(j)\right]}^{2}};p,q\in [1,N].$$

The SPXY algorithm is an extension of the KS algorithm, which takes into account both the x and y variables when calculating the distances between samples [44]. In the SPXY algorithm, the distances between samples are calculated by considering the simultaneous variations in both the x and y variables. This allows for a more comprehensive evaluation of the sample distances and provides a refined approach for sample selection in the training set.

#### 2.4.5. Model Construction

Partial least squares regression (PLS) is a modeling method used to fit multiple dependent variables to multiple independent variables. It is commonly employed in constructing linear regression models for spectral data and effectively addresses issues related to high collinearity in the spectral data. By evaluating the regression curve on a validation set, the method calculates the limit of detection (LOD) and limit of quantification (LOQ) for the model [45].

Support vector machine (SVM) is a powerful machine learning algorithm widely used in both classification (SVC) and regression analysis (SVR). It is known for its strong mathematical theoretical support, high interpretability, and independence from statistical methods. SVM is particularly suitable for handling small-batch samples.

#### 2.4.6. Reliability Verification of the Model

The determination coefficients (R^2^c and R^2^p) and root mean square errors (RMSEC, RMSEP) are used as model evaluation metrics, based on the training set and prediction set. The formulas for these calculations are provided in Equations (5) and (6). The determination coefficient (R^2^) is a statistical measure that reflects the correlation between the absorbance at specific wavelengths and the physicochemical properties of the samples. RMSEC is used to assess the fitting accuracy of the constructed model, while RMSEP is used to evaluate the predictive ability of the model for target samples [46]. A higher *R*^2^ value closer to 1 and a smaller RMSE indicate a better agreement between the measured and predicted values, indicating a more reliable model [47].

(5) $$R^{2}=1-\frac{\frac{1}{M}\sum_{i=1}^{m}{\left(f_{i}-y_{i}\right)}^{2}}{\frac{1}{m}\sum_{i=1}^{m}{\left({\bar{y}}_{i}-y_{i}\right)}^{2}}$$

(6) $$RMSE=\sqrt{\frac{1}{m}\sum_{i=1}^{m}{\left(f_{i}-y_{i}\right)}^{2}}$$

In the Equations provided: *f_i_* represents the true value of the *i*th sample, *y_i_* represents the predicted value of the *i*th sample, ${\bar{y}}_{i}$ represents the average value of all the true values of the samples, and *M* represents the number of samples.

### 2.5. Software and Programs

The data for this study were stored using Excel software (2016 edition, Microsoft Corporation, Washington, DC, USA). The variable selection, spectral preprocessing, outlier removal, and construction of prediction models were implemented using MATLAB software (2020 edition, MathWorks Corporation, Natick, MA, USA).

## 3. Results and Discussion

### 3.1. Textural Properties and TVB-N Value Analysis

**Figure 3:**
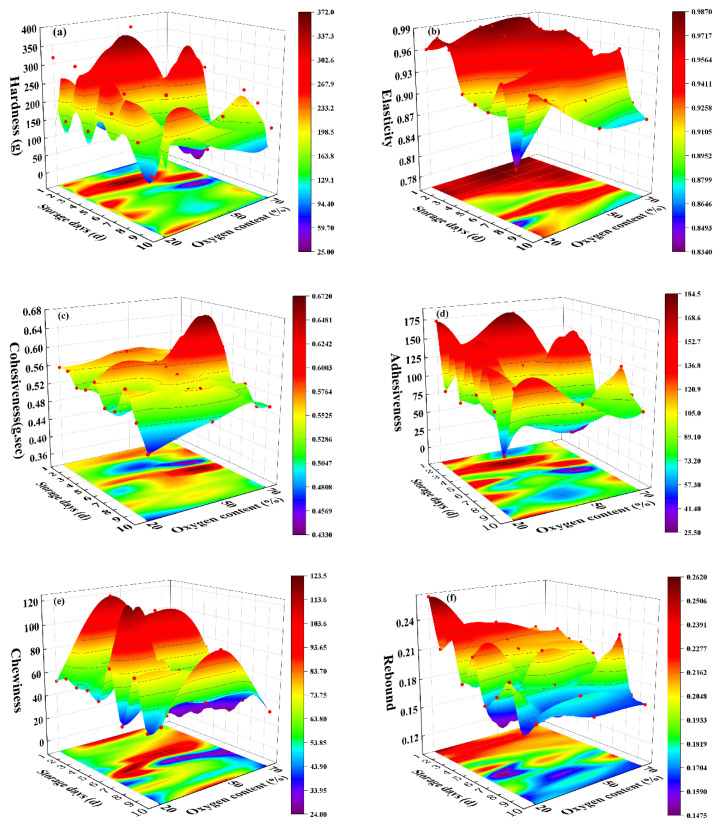
Variation of different textural parameters with storage conditions and time (4 °C): (**a**) hardness variation over time; (**b**) elasticity variation over time; (**c**) cohesiveness variation over time; (**d**) adhesiveness variation over time; (**e**) chewiness variation over time; (**f**) rebound variation over time.

**Figure 4:**
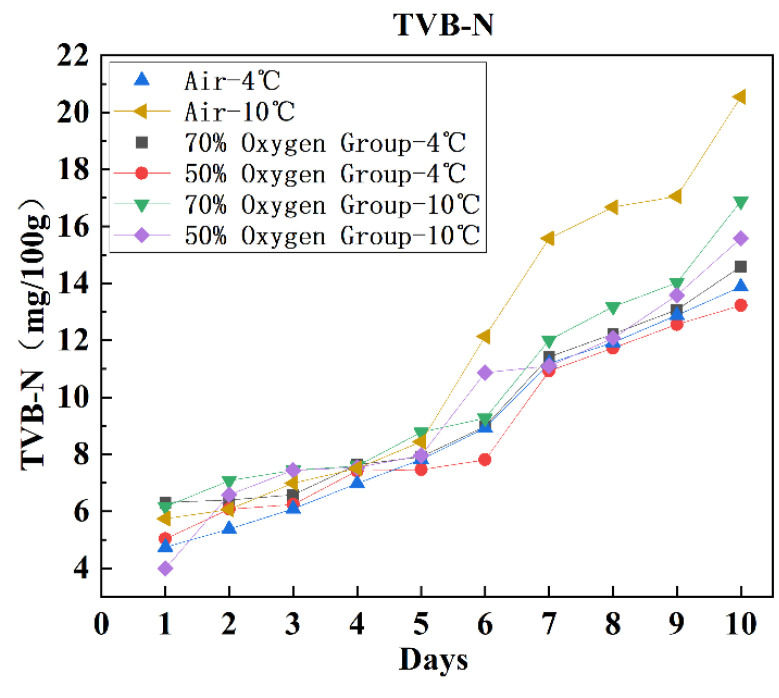
Curve of TVB-N content with environment and time.

The trends of the indicators under different lamb meat storage conditions over time are shown in Figure 3 and Figure 4. Hardness, elasticity, cohesiveness, adhesiveness, and chewiness did not show a clear increasing or decreasing trend over time, indicating significant fluctuations in the texture parameters. This suggests that there is considerable interference during measurement, leading to some level of data error. Therefore, it is necessary to select specific indicators to ensure the accuracy of the model. Based on modeling analysis, it was found that resilience and TVB-N exhibited higher prediction accuracy compared to other indicators. The prediction accuracy of the remaining indicators was below 0.6, with model errors exceeding the acceptable range. Hence, those indicators were discarded. Finally, rebound and TVB-N were selected as parameters for modeling.

As time increases, the noticeable decrease in rebound indicates a decline in lamb meat quality and a reduction in freshness. The decline in resilience is relatively slow from 0 to 5 days, but the rate of decline accelerates after the 6th day. This may be attributed to significant meat spoilage during the later stages of storage, resulting in substantial loss of moisture, extensive tissue damage, and enhanced bottom effect [48]. On the other hand, the TVB-N content in each group shows a significant increase with increasing storage time.

### 3.2. Spectral Preprocessing Results

To select the threshold range, partial least squares regression (PLS) is used to model and predict. The root means square error of cross-validation (RMSEC) is calculated, and the optimal weight coefficients and threshold are determined based on the minimum RMSEC. The corresponding outlier sample serial numbers are identified and removed. Similarly, using the same approach, the best spectral preprocessing methods for modeling with resilience and TVB-N as parameters are found to be the SG-2nd method and the MSC method.

**Figure 5:**
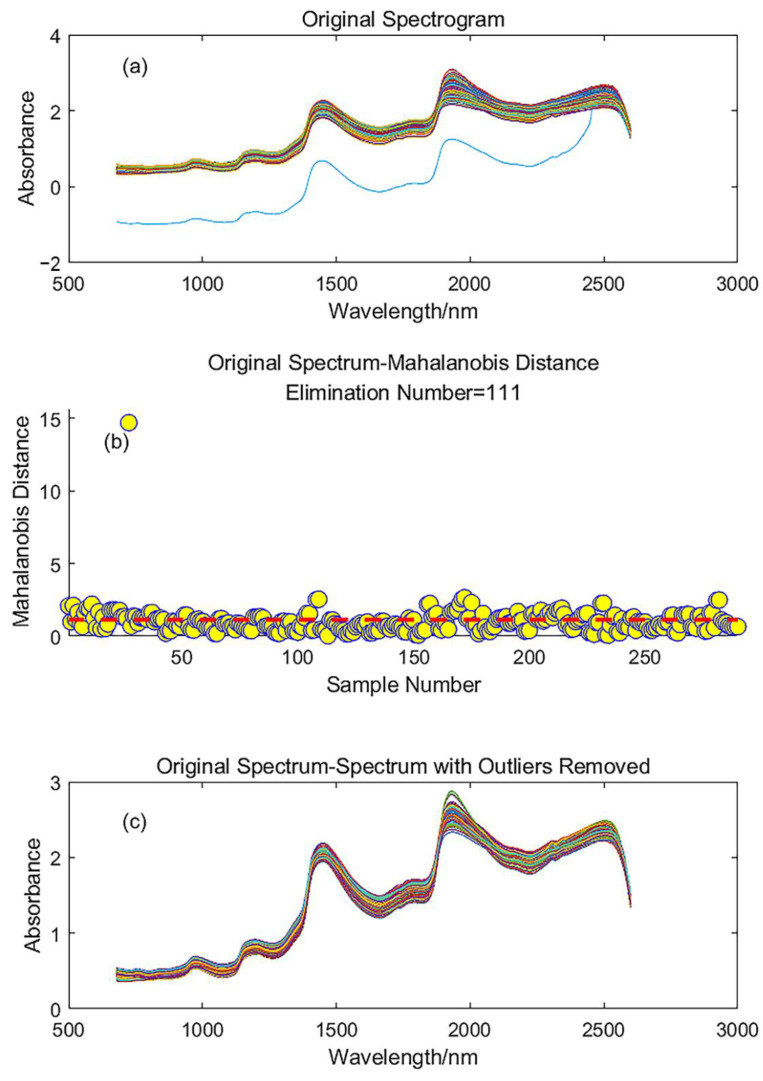
The original spectrum and the spectrum after removing outliers: (**a**) original spectrogram; (**b**) marginal distance rejection and optimal threshold; (**c**) spectrum after excluding abnormal samples.

If the original spectral data is directly subjected to Mahalanobis distance-based outlier removal, a total of 111 samples are removed, including severe outliers as shown in Figure 5. The red dashed line represents the threshold, and the area above the dashed line corresponds to the removed portion, while the area below represents the retained sample numbers. However, since the number of removed samples is relatively large compared to the original sample size, it is advisable to perform preprocessing first and then remove the outliers.

**Figure 6:**
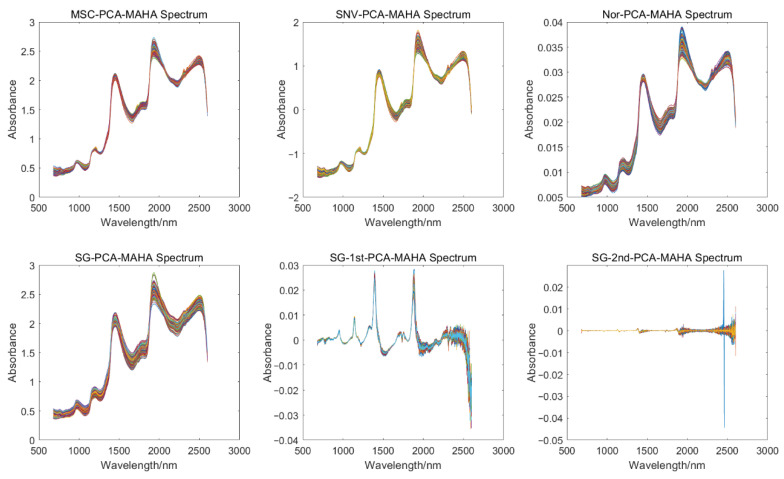
Spectrum after rejection of 6 preprocessed abnormal samples.

After applying the 6 preprocessing methods, the number of removed outliers varies for each method, as shown in Table 2. Among them, the SG-2nd preprocessing method resulted in the least number of removed outliers, with 7 samples, while the SG preprocessing method had the highest number of removed outliers, with 111 samples. The spectra after removing outliers for each preprocessing method are shown in Figure 6.

### 3.3. Results of Dividing the Sample Set

The experimental samples were divided into training and prediction sets using three different methods: random splitting, Kennard–Stone algorithm, and SPXY algorithm. Since the number of samples varies after combining each preprocessing method with outlier removal, the ratio of a training set to a prediction set was set to 7:3 for consistency.

To compare the effectiveness of different sample partitioning methods, R2 and RMSE were used as indicators to select the best partitioning method. According to Table 3, the prediction set R2 values for the KS algorithm and SPXY algorithm are the same, both at 0.6257. However, the SPXY algorithm has a lower RMSEP value compared to the KS algorithm. On the other hand, the random splitting method resulted in the prediction set R2 values ranging from 0.57 to 0.72, showing less stability compared to the SPXY algorithm. Additionally, the RMSEP values for random splitting were all higher than those of the SPXY algorithm. In summary, it is recommended to use the SPXY algorithm for data set partitioning and model building.

### 3.4. Feature Wavelength Selection Results

**Figure 7:**
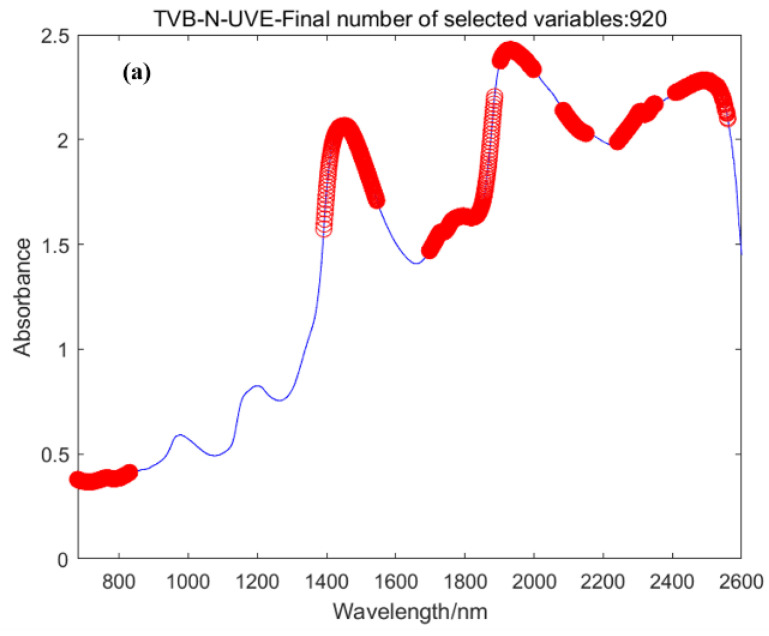

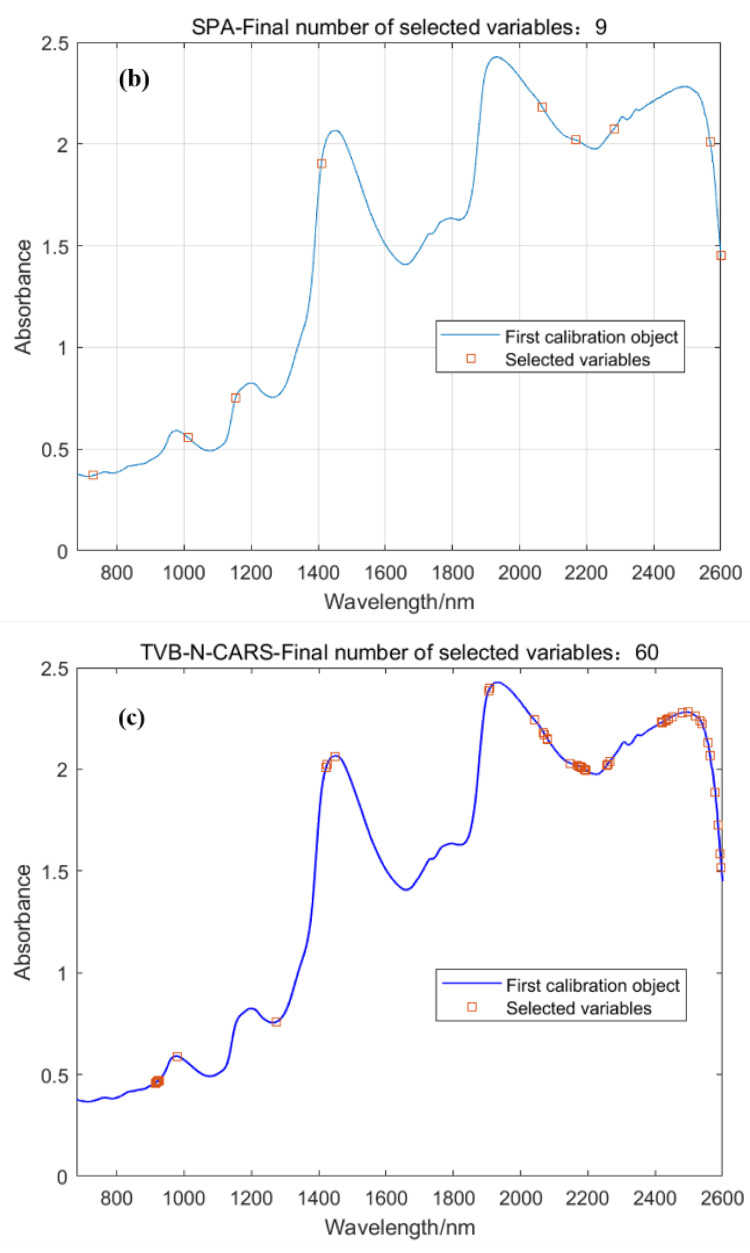
Feature wavelength selection results: (**a**) UVE; (**b**) SPA; (**c**) CARS.

The wavelength points for acquiring spectral data from the instrument are 1921. Within a large number of wavelength points, there are redundant spectral bands and noise. By eliminating irrelevant wavelengths and replacing them with characteristic wavelengths, the model can be simplified. Taking TVB-N as an example, the sequence number of wavelength points is obtained through the UVE method, and random noise is added to each curve corresponding to the sequence number of the wavelength points. A total of 920 characteristic wavelength points were selected. However, this method may result in too many characteristic wavelength points and potential collinearity issues. Therefore, the UVE method is more suitable for the rough selection of characteristic wavelength points. Based on the SPA method, 9 characteristic wavelength points were obtained, namely 729, 1012, 1154, 1409, 2069, 2167, 2283, 2568, and 2600. The CARS method determines the optimal subset based on the lowest root mean square error value from cross-validation and obtains 60 variables. The variable selection results from each method are shown in Figure 7.

### 3.5. Prediction Modeling

#### 3.5.1. Results of PLS Model Based on Characteristic Waveform

**Figure 8:**
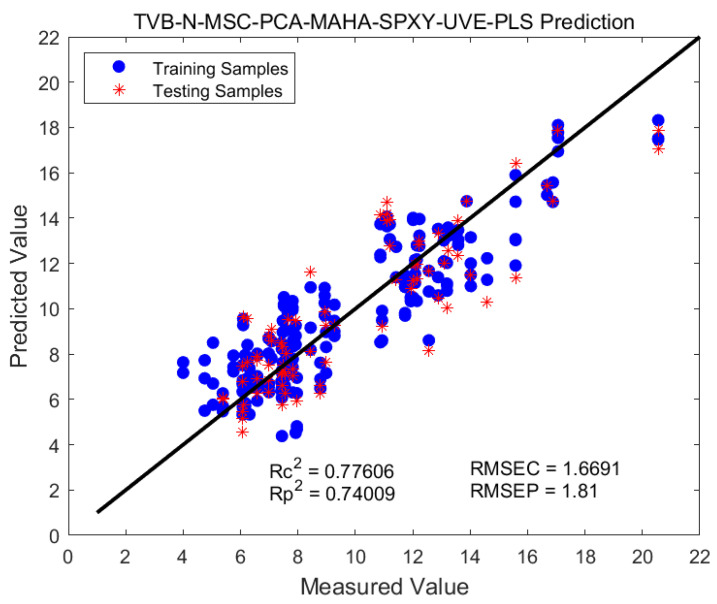
MSC-UVE-PLS model predicts TVB-N results.

The predictive modeling results for the response variable, based on the SG-2nd method for spectral preprocessing and incorporating three different feature wavelength treatments, are presented in Table 4. After the selection of feature wavelengths, the accuracy of the predictive model for the response variable significantly improved. Among them, the SG-2nd-SPA model showed the best performance (R^2^c = 0.95, R^2^p = 0.94). Modeling the TVB-N variable and comparing it with the full spectral range PLS model, all evaluation metrics improved when using the MSC combined with three feature wavelengths in the PLS model. The PLS model without any treatment yielded an effectiveness of 0.45, which improved to 0.62 after MSC preprocessing. Furthermore, after wavelength treatment, the model accuracy reached 0.7 to 0.75, with the UVE method showing the highest accuracy of 0.74, corresponding to an RMSEP of 1.81. This effectively enhanced the model’s accuracy. The prediction results of the MSC-UVE-PLS model for TVB-N are depicted in Figure 8.

#### 3.5.2. Results of the SVM Model Based on the Characteristic Waveform

**Figure 9:**
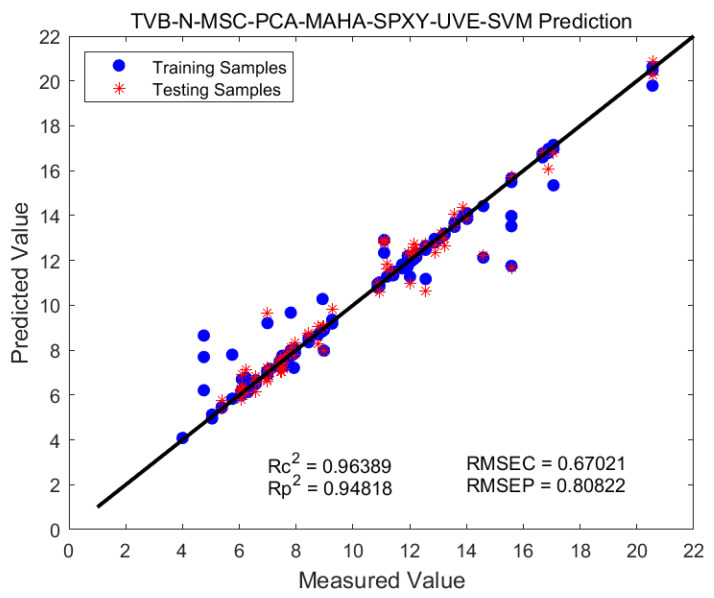
MSC-UVE-SVM model predicts TVB-N results.

According to Table 5, the SVM model demonstrates good predictive performance for both indicators. The SG-2nd-SVM and MSC-SVM models improve the model’s performance by 0.3 to 0.4. Building upon this, when combined with SPA and UVE wavelength treatments, there is a slight further improvement in model performance. Regarding the response indicator, the SG-2nd-SPA model shows the best performance (R^2^c = 0.94, R^2^p = 0.90). However, for predicting TVB-N content, the SVM models, after wavelength selection using the CARS and SPA algorithms, exhibit lower accuracy compared to before the treatment. Hence, the optimal approach is MSC-UVE. Figure 9 illustrates the prediction results of the MSC-UVE-SVM model for TVB-N.

### 3.6. Comparison of PLS and SVM Models

**Figure 10:**
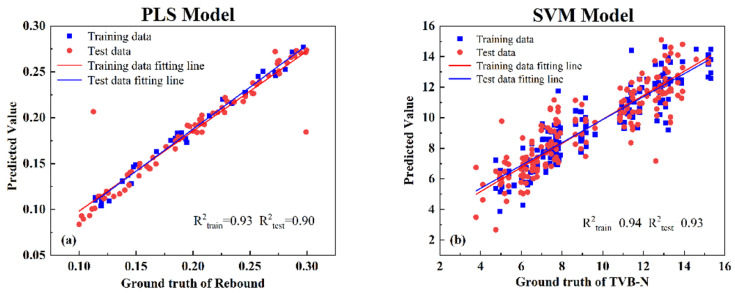
Out-of-sample validation: (**a**) rebound-PLS model; (**b**) TVB-N-SVM model.

Both models show consistent performance for predicting the rebound variable, with the optimal model obtained from the linear PLS model (SG-2nd-SPA-PLS). However, for predicting TVB-N content, the SVM model is more suitable. In a longitudinal comparison with the PLS model, the SVM model outperforms it, with higher R^2^ values for both the test and prediction sets. The MSC-UVE-SVM model shows a strong linear correlation between predicted and actual values, indicating its superior performance and making it the best model. To further validate the developed models, three new samples were tested, including pure lamb meat, pure chicken meat, and a 50% chicken–lamb meat mixture. The results are shown in Figure 10, and they demonstrate good performance. The PLS model based on the response variable achieved an R^2^_train_ of 0.93 and an R^2^_test_ of 0.90, while the SVM model based on TVB-N content achieved an R^2^_train_ of 0.94 and an R^2^_test_ of 0.93. These results indicate that linear PLS regression performs well for predicting target values with small variations, while SVM is a modeling method suitable for small-sample situations with large variations in the target values.

## 4. Conclusions

This study employed the parameters of response and TVB-N to comprehensively evaluate the quality of modified atmosphere-packaged lamb meat and developed a prediction model based on near-infrared spectroscopy. The results revealed that when using the response parameter, the SPA-PLS model based on second derivative preprocessing achieved the highest accuracy, with an R^2^p of 0.9383 for the prediction set. On the other hand, when using TVB-N as the parameter, the UVE-SVM model based on MSC preprocessing demonstrated the best performance, with a model prediction R^2^p of 0.9482. The study findings demonstrate that the combination of near-infrared spectroscopy and chemometrics significantly improves the prediction performance of TVB-N content in lamb meat and achieves rapid non-destructive detection. In terms of model performance, the linear regression model PLS is more suitable for regression prediction problems with smaller variations in the target value compared to the SVM model. Integrating machine learning algorithms to develop regression prediction models can better exploit the information in near-infrared spectroscopy. The developed method offers advantages such as simple preparation, high throughput, and good performance, which will contribute to the quality and safety control of meat products in the food industry. It also provides a theoretical basis and technical support for the development of portable near-infrared spectrometers for the rapid freshness determination of lamb meat.

## Figures and Tables

**Table 1:** Storage of lamb in 6 groups.

Group	Temperature (°C)	O_2_ (%)	CO_2_ (%)	N_2_ (%)
A	4	70	20	10
B	4	50	40	10
C	4	Air	Air	Air
D	10	70	20	10
E	10	50	40	10
F	10	Air	Air	Air

**Table 2:** Number of abnormal samples rejected by 6 spectral preprocessing methods.

Preprocessing Methods	Number of Abnormal Rejection Samples
MSC *	29
SNV	28
Nor	15
SG	111
SG-1st	109
SG-2nd	7

* MSC, multi-scatter calibration; SNV, standard normal variate transformation; Nor, normalization; SG, Savitzky–Golay smoothing; SG-1st, SG first-order derivative; SG-2nd, SG second-order derivative.

**Table 3:** Model prediction results under 4 sample division methods (TVB-N).

Classification Method	Number of Principal Components	Training Set		Prediction Set	
		R^2^c *	RMSEC	R^2^p	RMSEP
Random division	10	0.7706	1.6311	0.7199	2.0226
	10	0.7835	1.6952	0.6581	1.8747
	8	0.7161	1.8673	0.7185	1.9128
	10	0.7188	1.5935	0.7139	1.9707
	10	0.8104	1.5934	0.5797	2.0873
KS	10	0.7029	1.7040	0.6257	1.8912
SPXY	9	0.6552	1.8733	0.6257	1.7972

* R^2^c, training set determination factor; R^2^p, prediction set decision factor; RMSEC, training set root mean square error; RESEP, prediction set root mean square error; KS, Kennard–Stone; SPXY, sample set partitioning based on joint X-Y distance.

**Table 4:** PLS model prediction results for 2 indicators.

Physical and Chemical Indicators	Preprocessing Methods	Number of Principal Components	Training Set Results		Prediction Set Results			
			R^2^c *	RMSEC	R^2^p	RMSEP	LOD	LOQ
Rebound	None	10	0.63	1.03	0.46	1.26	56.99	130.85
	SG-2nd	9	0.90	0.06	0.886	0.07	120.82	370.83
	SG-2nd-CARS	9	0.93	0.05	0.91	0.06	12.81	45.36
	SG-2nd-UVE	8	0.94	0.05	0.91	0.06	13.56	47.42
	SG-2nd-SPA	7	0.95	0.04	0.94	0.05	15.93	53.65
TVB-N	None	10	0.63	1.03	0.45	1.38	10.69	33.54
	MSC	9	0.65	1.87	0.63	1.79	46.99	120.36
	MSC-CARS	9	0.78	1.64	0.73	1.82	46.98	140.94
	MSC-UVE	10	0.78	1.66	0.74	1.81	126.45	374.65
	MSC-SPA	9	0.71	1.89	0.72	1.89	13.26	80.83

* R^2^c, training set determination factor; R^2^p, prediction set decision factor; RMSEC, training set root mean square error; RESEP, prediction set root mean square error; none, raw spectrum without using any preprocessing technique; SG-2nd, SG second-order derivative; MSC, multi-scatter calibration; CARS, competitive adaptive reweighted sampling; SPA, successive projections algorithm; UVE, uninformative variable elimination; LOD, limit of detection; LOQ, limit of quantification.

**Table 5:** SVM model prediction results for 2 metrics.

Physical and Chemical Indicators	Preprocessing Methods	Wave Length Point	Training Set Results		Prediction Set Results			
			R^2^c *	RMSEC	R^2^p	RMSEP	LOD	LOQ
Rebound	None	1921	0.60	0.14	0.58	0.16	13.67	35.62
	SG-2nd	1921	0.86	0.07	0.83	0.08	46.96	130.25
	SG-2nd-CARS	87	0.90	0.06	0.86	0.07	33.87	101.60
	SG-2nd-UVE	1034	0.91	0.05	0.87	0.07	27.20	81.61
	SG-2nd-SPA	14	0.94	0.05	0.90	0.06	37.18	111.54
TVB-N	None	1921	0.70	0.92	0.59	1.19	27.70	86.16
	MSC	1921	0.94	0.12	0.93	0.75	276.56	812.62
	MSC-CARS	60	0.92	0.98	0.88	1.22	16.36	46.98
	MSC-UVE	920	0.96	0.67	0.95	0.80	16.35	49.38
	MSC-SPA	9	0.89	1.16	0.87	1.28	120.34	315.32

* R^2^c, training set determination factor; R^2^p, prediction set decision factor; RMSEC, training set root mean square error; RESEP, prediction set root mean square error; none, raw spectrum without using any preprocessing technique; SG-2nd, SG second order derivative; MSC, multi-scatter calibration; CARS, competitive adaptive reweighted sampling; SPA, successive projections slgorithm; UVE, uninformative variable elimination; LOD, limit of detection; LOQ, limit of quantification.

## Data Availability

The data presented in this study are available on request from the corresponding author. The data are not publicly available due to copyright implications.
